# Factors Affecting Transcranial Motor-Evoked Potential Measurements Using Single-Train Stimulation with an Increased Number of Pulses during Adolescent Scoliosis Surgery: A Prospective Observational Study

**DOI:** 10.3390/jcm12134433

**Published:** 2023-06-30

**Authors:** Takayuki Toki, Noriaki Fujita, Tomohiro Ichikawa, Noriki Ochi, Isao Yokota, Hideki Sudo, Yuji Morimoto

**Affiliations:** 1 Department of Anesthesiology, Hokkaido University Hospital, N14W5, Sapporo 060-8648, Japan; fujitan@mac.com (N.F.); morim2@med.hokudai.ac.jp (Y.M.); 2 Division of Laboratory and Transfusion Medicine, Hokkaido University Hospital, N14W5, Sapporo 060-8648, Japan; tomo090765@gmail.com (T.I.); och-non@huhp.hokudai.ac.jp (N.O.); 3 Department of Biostatistics, Graduate School of Medicine, Hokkaido University, Sapporo 060-8638, Japan; yokotai@pop.med.hokudai.ac.jp; 4 Department of Orthopedic Surgery, Hokkaido University Hospital, N14W5, Sapporo 060-8648, Japan; hidekisudo@yahoo.co.jp

**Keywords:** transcranial motor-evoked potentials, adolescent scoliosis, intraoperative neurological monitoring

## Abstract

Measurement of transcranial motor-evoked potentials (TcMEPs) during scoliosis surgery helps detect postoperative new neurological defects. However, TcMEP interpretation is difficult owing to the influence of intraoperative physiological, pharmacological, and time-related factors as well as stimulation conditions. In this study, we aimed to investigate the effect of the abovementioned factors on TcMEP amplitude using single-train stimulation with an increased number of pulses (STS-INP) during adolescent scoliosis surgery; moreover, we evaluated the complications of TcMEP measurement. We included 50 patients and 706 TcMEP measurements. A total of 1412 TcMEP waveforms were analyzed, each on the bilateral abductor pollicis brevis, tibialis anterior, and abductor hallucis muscles. We estimated the mean difference (95% confidence interval (CI)) and predicted mean difference (95% CI) evaluated using the interquartile range of each factor, based on a mixed-effect model with random intercepts for TcMEP amplitude. The predicted mean differences in TcMEP amplitude were clinically small compared with the actual TcMEP amplitude, suggesting that each factor had a limited effect on TcMEP amplitude. No intraoperative bite injuries or seizures were observed. Using STS-INP during adolescent scoliosis surgery may enable accurate measurement of TcMEP amplitude with neither complications nor the influence of various intraoperative factors.

## 1. Introduction

Scoliosis is the most common pediatric musculoskeletal disease, with a prevalence of 0.3–15.3% [1]. Surgical correction is considered in patients having progressive scoliosis with a Cobb angle greater than 40–45° [2]. New neurological defects (NNDs) complicate approximately 1.0% of spinal deformity surgeries that are performed for scoliosis [3,4]. The determination of transcranial motor-evoked potentials (TcMEPs) during scoliosis surgery has been reported to be a sensitive and specific method for detecting postoperative NNDs [5]. Additionally, intraoperative neurophysiologic monitoring, including TcMEP determination, reportedly reduces the incidence of NNDs [6].

However, the immaturity of the central nervous system in children complicates TcMEP monitoring or interpretation [7,8]. In addition, intraoperative factors such as anesthetic agent use, neuromuscular blocker use, blood pressure, temperature, oxygenation, and operation time have been reported to affect TcMEP [9]. Therefore, a higher number of pulses may be required to obtain stable TcMEP amplitudes in pediatric patients [7]. However, TcMEPs are generally measured using a train of 3–6 pulses [8,9]. We attempted to stabilize the amplitude of TcMEPs during adolescent scoliosis surgery by using single-train stimulation (STS) with an increased number of pulses (6–15 pulsed, STS-INP) to obtain the maximum TcMEP amplitude. TcMEP measurements during adolescent scoliosis surgery using STS-INPs have not been reported, and the effect of intraoperative confounding factors on TcMEP amplitude is unknown. In addition, TcMEP measurements may induce seizures and bite injuries of the tongue and oral cavity [8,9,10,11]; however, the occurrence of these adverse events with STS-INP remains unclear.

Therefore, in this study, we aimed to investigate the intraoperative physiological, pharmacological, and time-related effects of STS-INP on TcMEP amplitude during adolescent scoliosis surgery and to investigate the occurrence of adverse events.

## 2. Materials and Methods

### 2.1. Study Design and Ethics

**Figure 1:**
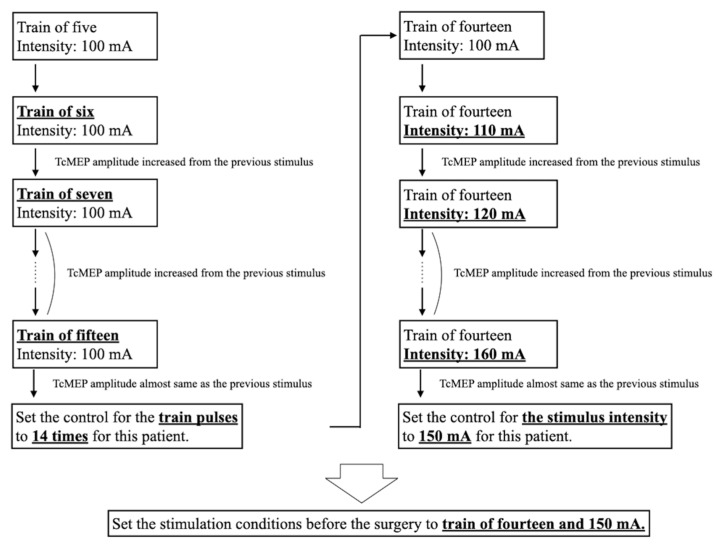
Typical process of setting TcMEP stimulation conditions. First, we examined the number of pulses that produced the maximum amplitude. Subsequently, we searched for the stimulus intensity that produced the maximum amplitude, with the maximum stimulation output of 200 mA. Bold and underlined text indicates changes in stimulation conditions or the final setting. TcMEP—transcranial motor-evoked potential.

This study was a single-center, prospective observational study. The sample group is explained in Section 2.1, Study design and ethics; the study plan and interventions are explained in Figure 1; and the sample selection and size are stated in Section 2.4, Rationale for Number of Cases. This study was approved by the Ethics Committee of the Hokkaido University Hospital. All methods were implemented according to relevant guidelines and regulations. Written informed consent was obtained from all the patients and their guardians. From July 2019 to August 2021, we conducted a prospective observational study in patients aged 10–19 years who underwent initial spinal deformity correction surgery using posterior approach for adolescent scoliosis at Hokkaido University Hospital. We excluded the following patients: (1) those who did not undergo initial surgery, (2) those who used inhaled anesthetic to maintain general anesthesia, (3) those with American Society of Anesthesiologists Physical Status class ≥ III, and (4) those with preoperative neuromuscular disease.

### 2.2. Anesthesia and Monitoring Technique

No premedication was administered. After entering the operating room, patients underwent electrocardiogram, blood pressure, and percutaneous arterial blood oxygen saturation monitoring.

General anesthesia was induced using either a propofol target-controlled infusion (Terufusion™ Syringe pump, TERUMO, Tokyo, Japan), a rapid injection of propofol, or slow induction with sevoflurane. Moreover, fentanyl, remifentanil, and rocuronium were administered. After tracheal intubation, ventilation was controlled at a tidal volume of 6–8 mL/kg. The respiratory rate was adjusted to maintain a carbon dioxide end-tidal concentration of 35–40 mmHg.

Arterial blood pressure was measured directly at the radial artery. Total intravenous anesthesia was maintained with propofol, fentanyl, and remifentanil. The propofol dose was adjusted to achieve a bispectral index (BIS; BIS™ monitor, Covidien, Minneapolis, MN, USA) of 40–60. Neuromuscular blockers were not administered after intubation. The intraoperative core temperature of the bladder was continuously monitored and maintained at 36–37 °C, when possible. In addition, a bite block covered with gauze was placed in the oral cavity to prevent injuries associated with TcMEP measurements [12].

Propofol and remifentanil infusions were discontinued at the end of surgery. The tracheal tube was removed after the patient awoke and resumed spontaneous ventilation. A single team of orthopedic surgeons performed all surgeries.

### 2.3. TcMEP Measurements

We used MEE-1216 (Nihon Koden, Tokyo, Japan) and MS-120B (Nihon Koden, Tokyo, Japan) for the TcMEP measurements and stimulation, respectively. TcMEPs were generated by placing stimulating dish electrodes on a 2-cm area on the ventral scalp of C3 and C4 using the international 10–20 electroencephalogram system. Needle electrodes were inserted into the bilateral abductor pollicis brevis muscles (APB), tibialis anterior muscles (TAM), and abductor hallucis muscles (AHM). We used STS with bi-phasic stimulation at an intensity of 100–200 mA, a train of 6–18 pulses, intervals of 2 ms, and a pulse duration of 0.5 ms. The loss of muscle relaxation was defined by the laboratory technician in charge of TcMEP measurement after a sufficient duration from the last muscle relaxant administration and a non-significant change in the amplitude after multiple TcMEP stimulations under the same conditions and at different time intervals. To obtain a maximum amplitude with the maximum stimulation output of 200 mA possible for the MS-120B, we established the number of pulses and the stimulus intensity. Before scoliosis correction, the maximum amplitude was recorded under the abovementioned conditions as the control value (Figure 1). During scoliosis correction, TcMEP measurement was initiated simultaneously with the first screw implantation and was performed at each screw implantation. Intraoperatively, the technician adjusted the number of pulses and the stimulus intensity when the TcMEP amplitudes of the extremities changed uniformly.

### 2.4. Rationale for the Number of Cases

To date, no data has been published on TcMEP measurements during adolescent scoliosis surgery using STS-INP. Therefore, we could not conduct a priori sample size calculations for this study because the effect size could not be estimated. The study aimed to recruit 50 participants; we expected to achieve this sample size within a reasonable time based on study recruitment rates within our hospital. In 2018, 30 initial corrective surgeries were performed for adolescent scoliosis at our institute. Hence, we determined that 50 patients could be treated within a 25-month study period.

### 2.5. Statistical Analysis

Continuous variables were expressed as medians [interquartile ranges, IQRs] which does not require the assumption of a distribution, and categorical variables were expressed as numbers and percentages. Since the nerve myelination process continues from childhood through adolescence [13], we evaluated the required number of pulses and the stimulus intensity after dividing participants into two age groups: early (age 10–14 years) and late (age 15–19 years) teenage groups. The mean difference and its 95% confidence interval (CI) of the TcMEP amplitude were evaluated using a mixed-effect model including the following parameters: BIS [14], mean arterial pressure (MAP) [9,15,16], oxygen saturation [17], core temperature [18,19], estimated blood propofol concentration [20,21,22], surgical elapsed time at TcMEP stimulation (time elapsed from the start of surgery, measured to account for the anesthesia fade phenomenon) [9,23], number of train pulses, and stimulation intensity. A random intercept of patients was included to take into account the repeated measurements within the same patients. Furthermore, we estimated the predicted mean difference and its CI when the level of each factor was changed between the IQR to interpret them as the magnitude of the clinically plausible change. Hence, this study used a mixed-effects model and assessed IQR; it was not based on *p*-values. We did not include the estimated propofol effect-site concentration in the model because of its strong correlation with the estimated blood propofol concentration. Intraoperative opioid use and predicted blood concentrations of opioids were not included because of their lack of effect on intraoperative neuromonitoring [16]. In addition, we examined the incidence of intraoperative bite injuries, seizures, and postoperative lower extremity NNDs. R version 4.2.2: Language and environment for statistical computing (R Foundation for Statistical Computing, Vienna, Austria at https://www.R-project.org/ (accessed on 10 January 2023)) was used to create charts, to summarize the data, and for statistical analysis.

## 3. Results

### 3.1. Patient Demographic, Perioperative, and TcMEP Measurement Data

**Figure 2:**
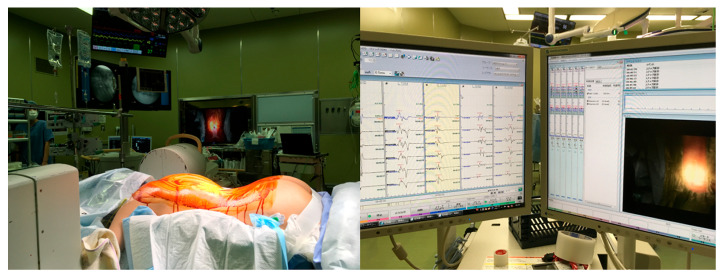
A picture with example of monitoring a patient.

During the case enrollment period, 53 patients underwent an initial surgery for adolescent scoliosis (Figure 2). The following were excluded: one patient who could not be included because of the absence of a researcher on the day of surgery, one patient who was allergic to propofol, and one patient who did not undergo BIS measurement. Consequently, 50 patients were included in the analysis. The median patient age was 14 [IQR: 13, 17] years, with a predominance of female participants (82%); moreover, idiopathic scoliosis was the most common type of scoliosis, with an incidence of 82% (Table 1). The median total operative time was 252 [IQR: 212, 283] min (Table 1).

**Figure 3:**
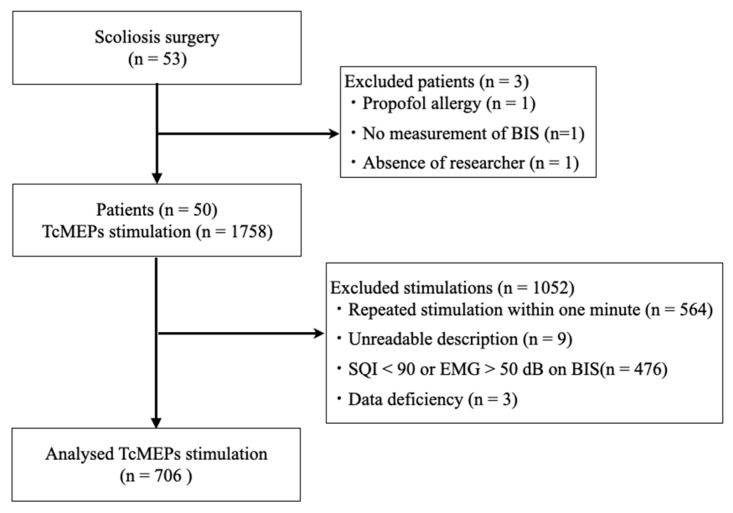
Patient selection and TcMEP measurement flow chart. TcMEP—transcranial motor-evoked potential; BIS—bispectral index; SQI—signal quality index; EMG—electromyogram.

A total of 1758 TcMEP measurements were performed in these patients, 706 of which were included in the study, after excluding 564, 9, 476, and 3 measurements performed within 1 min due to illegible recording forms, a signal quality index < 90, electromyogram activity > 50 on BIS, and incomplete data, respectively (Figure 3).

### 3.2. TcMEP Parameters

**Figure 4:**
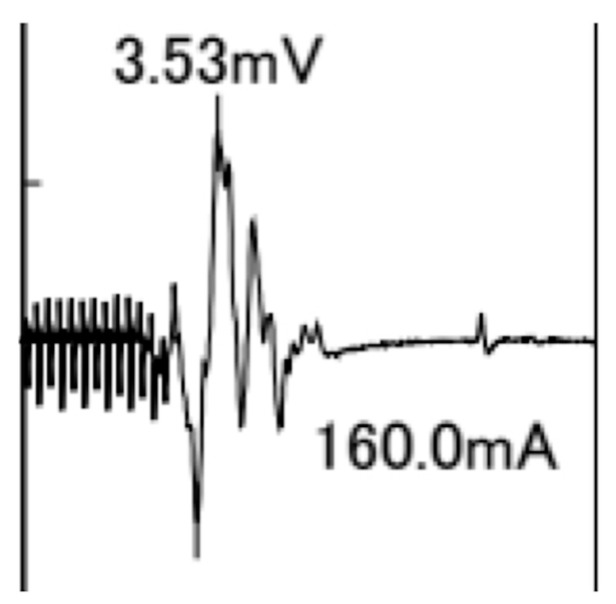
Typical transcranial motor-evoked potential waveform for the abductor pollicis brevis muscle with 13 pulse stimuli and an intensity of 160 mA. The amplitude is 3.53 mV.

A typical TcMEP waveform for the APB is shown in Figure 4.

Table 2 presents the parameters for all 706 TcMEP measurements. A total of 1412 bilateral TcMEP waveforms were analyzed for the APB, TAM, and AHM. The median amplitudes of the APB, TAM, and AHM were 3.25 mV [IQR: 2.50, 4.18 mV], 1.36 mV [IQR: 0.89, 1.91 mV], and 1.96 mV [IQR: 1.39, 2.70 mV], respectively. The time elapsed from muscle relaxant administration during each TcMEP stimulation ranged from 78 to 442 min. The medians of required pulses for participants aged 10–14 and 15–19 years were 13 [IQR: 12, 15] (range: 7–17) and 12 [IQR: 11, 13] (range: 9–17), respectively. The medians of required stimulus intensities for participants aged 10–14 and 15–19 years were 150 [IQR: 140, 170] and 140 [IQR: 130, 150] mA, respectively. The number of required pulses and the stimulus intensity decreased as age group increased. In summary, the number of pulses was greater, and the stimulus intensity was either the same or less than that documented in previous reports (Table 3).

### 3.3. Influence of Physiological, Pharmacological, and Time-Related Factors as Well as Stimulation Conditions on TcMEP Amplitude

Table 4 presents the intraoperative factors affecting TcMEPs at the time of stimulation. The median of MAP was 55 mmHg [IQR: 51, 61 mmHg], percutaneous oxygen saturation was 100% [IQR: 100, 100%], core temperature was 36.7 °C [IQR: 36.4, 37.0 °C], pulse rate was 78 waves/min [IQR: 69, 85 waves/min], BIS was 44 [IQR: 39, 49], estimated blood concentration of propofol was 3.40 µg/mL [IQR: 3.00, 3.90 µg/mL], and surgical elapsed time at TcMEP stimulation was 144 min [IQR: 98, 199 min].

Table 5 shows the mean differences (95% CI) and the predicted mean differences (95% CI) evaluated using the IQR of each factor, based on the mixed-effect model with random intercepts for TcMEP amplitude. The largest absolute of predicted changes in perioperative physiological, pharmacological, and time-related factors as well as stimulation conditions were 0.456, 0.413, and 0.420 mV for the APB, TAM, and AHM, respectively. These values constituted only 14%, 30%, and 21% change in the median amplitude of each of the muscles, respectively. Previous reports have defined the alarm criterion for TcMEP in spinal surgery as an amplitude decline of 50–90% [8]. Clinically, these changes in median amplitude mean that surgeons should not concerned about NNDs.

### 3.4. NND and Complications of STS-INP

There was no critical decline in lower limb TcMEP amplitude in any patient, and no patient had any intraoperative bite injury, seizure, or lower limb NND.

## 4. Discussion

This study evaluated TcMEPs using STS-INP during the initial corrective surgery for pediatric and adolescent scoliosis. The predicted changes in each perioperative factor were small with respect to the actual TcMEP amplitude, suggesting that these changes did not significantly affect the TcMEP amplitude. Additionally, no adverse events, such as seizures or bite injuries, were observed during TcMEP measurements using STS-INP.

Intraoperative TcMEP changes in patients with idiopathic scoliosis can be used to detect new neuropathies with an average sensitivity of 91% (95% CI: 34–100%) and a specificity of 96% (95% CI: 92–98%) [5]. While the definition of warning criteria in TcMEP amplitude varies, most facilities define it as a decline of 50% or more [28,29]. The TcMEP amplitude is known to be affected by blood pressure, body temperature, depth of sedation, oxygenation, general anesthetic use, and surgical elapsed time at TcMEP stimulation, other than direct surgical damage [9]. An intraoperative MAP of 50–150 mmHg is required for TcMEP measurements to allow for cerebral blood flow autoregulation [16]. However, the MAP required for sufficient spinal cord blood flow remains unclear [16]. In a previous study on pediatric patients in whom the TcMEP waveform was lost during spine surgery, TcMEP amplitudes were restored to baseline values when MAP was increased from 68 mmHg to 86 mmHg, suggesting that MAP should be maintained above 85 mmHg as an initial response to critical decline in TcMEP amplitude [30]. Although the median MAP was low (55 mmHg) in this study, the decrease in MAP within the quartile range was not clinically significant.

Deep anesthesia also decreases the TcMEP amplitude [9]. A previous study evaluated the relationship between the BIS and motor-evoked potential (MEP) amplitude via MEPs elicited by 865 cortical stimulations in 28 patients undergoing awake craniotomy under propofol administration; the correlation coefficient between the BIS and MEP amplitude was 0.541, demonstrating that the MEP amplitude was significantly correlated with anesthesia depth [14]. However, a simple comparison between our findings and those of the abovementioned study would be difficult because direct cortical stimulation was applied in the study, which is different from the stimulation method used in our study. Indeed, the association between the BIS and TcMEP amplitude has not been evaluated in spinal surgery. In our study, changes in the BIS did not lead to clinically significant changes in the TcMEP amplitude. Propofol use reportedly decreases TcMEP amplitude in a blood concentration-dependent manner [20,21,22]. In our study, only a maximum TcMEP amplitude decrease of 263–456 µV was predicted within the estimated propofol blood concentration interquartile range. Conventionally, the number of pulses can be kept constant in all patients by setting the target TcMEP amplitude at 50–200 µV [25,31]. Because STS-INP produces high TcMEP amplitudes, blood propofol concentration does not seem to decrease the TcMEP amplitude in a clinically significant manner.

Extreme hypothermia is known to cause loss of TcMEPs [8,16]. Many studies examining the relationship between body temperature and TcMEP amplitude in cardiovascular surgery with hypothermia and circulatory arrest have reported that TcMEPs are restored at 34–35 °C [18,19]. In addition, TcMEP amplitude did not change up to a core temperature of 28 °C when train stimuli were used in animal experiments [32]. TcMEP amplitude only decreases in patients with severe hypothermia. Therefore, the effect of patient temperature was minor within the range of body temperatures measured in this study. In addition, the anesthetic fade phenomenon, in which the TcMEP amplitude decreases with time from TcMEP measurement initiation [9], could cause false positive reductions in the TcMEP amplitude [23]. Although the reason for anesthetic fade is not apparent, our findings demonstrated a small effect of the anesthetic fade phenomenon on TcMEP measurements using STS-INP. As described above, TcMEP amplitude measurement involves numerous intraoperative confounding factors; nevertheless, our findings indicate that STS-INP use may eliminate the effect of these factors on TcMEP amplitude measurement.

In adults, TcMEP measurements are generally performed in trains of four to six pulses [8]. A position statement by the American Society of Neurophysiological Monitoring indicated that a train of at least three pulses is necessary to generate MEPs [9]. However, a higher number of pulses may be required to obtain stable TcMEPs in pediatric patients owing to their immature central nervous system [7,8,9]. Most previous studies on scoliosis surgery used a constant number of five to seven pulses for continuous stimulation [23,24,25,26,27]. In our study, the number of required pulses and stimulus intensity decreased as age group increased. This indicates that the required number of pulses may differ among adolescent patients with scoliosis based on the level of myelination. Nevertheless, our study revealed that the effects of the number of pulses and stimulus intensity on the TcMEP amplitude were small. Thus, setting the number of pulses and stimulus intensity to obtain the maximum TcMEP amplitude in each case may be clinically useful, as it eliminates the influence of physiological, pharmacological, and time-related factors during general anesthesia, thereby providing a stable and accurate TcMEP amplitude.

The incidence of perioperative seizures associated with TcMEP measurements is reported to be 0.03–0.8% [8,9,33]. A retrospective study reported an absence of seizures or a low risk of seizures resulting from intraoperative TcMEP measurement in 18,862 patients who underwent spinal surgery [10]. In the current study, the patients did not develop seizures; hence, it is unlikely that STS-INP would increase the risk of developing perioperative seizures. Bite injuries are well-known complications of TcMEP measurement [12,33,34]. A retrospective study reported that bite injuries occurred in 0.63% of 17,273 patients who underwent TcMEP measurement [34]. Another retrospective study of 186 patients reported that bite injuries occurred in 6.5% of the study patients and cited severe body movements during TcMEP stimulation as a risk factor for bite injuries [12]. The same study also reported no difference in bite injury risk owing to stimulation settings such as stimulus intensity or tetanic stimulation [12]. In this study, no bite injury occurred in the 50 included patients; in addition, the incidence of bite injuries during TcMEP measurements using STS-INP was comparable to that during the conventional measurement method, in which the number of pulses is constant and lower than applied using our measurement method [34].

The present study provides valuable insight into TcMEP measurements during adolescent scoliosis surgery. This was the first prospective, multivariate study to report TcMEP measurement using STS-INP. As long as the STS-INP method is used, there are no intraoperative confounding factors on TcMEP amplitude. However, this study had some limitations. First, we could not confirm whether TcMEPs measured using STS-INP could detect early nerve injury because none of the patients in this study had postoperative NNDs. This study aimed to determine whether STS-INP provides a stable waveform or one affected by various factors. Changes during correction are almost never critically monitored in patients displaying moderate scoliosis. This problem can be important for spinal deformity surgery for curvature > 70°. Therefore, future studies should evaluate the clinical utility of TcMEP measurement using STS-INP. Second, our study findings were not directly compared with those obtained using conventional methods, such as adopting a lower number of pulses; hence, the superiority of our methods cannot be hitherto ascertained. Third, blood sampling tests could not be performed because this was an observational study; therefore, the blood concentration of propofol was predicted using a formula and may have differed from the actual concentration. Fourth, the possibility that the lower extremity amplitudes were influenced by surgical manipulation cannot be completely excluded. However, since no patient presented with postoperative NNDs, it is likely that there was no decrease in TcMEP amplitude associated with the surgical manipulation in this study. We believe that the parameters obtained from the lower extremity were reliable. Lastly, the residual muscle relaxant effects of rocuronium were not observed using a muscle relaxant monitor. Nevertheless, we believe that the possibility of residual muscle relaxant effects was considerably low, as sufficient time (at least 78 min) had passed since the last muscle relaxant administration and the TcMEP amplitude did not change significantly after multiple TcMEP stimulations under the same conditions and at different time intervals.

## 5. Conclusions

Because a higher number of pulses may be required to obtain stable TcMEP amplitudes in pediatric patients, this study evaluated TcMEPs using STS-INP during initial corrective scoliosis surgery. The predicted changes in each perioperative factor were small with respect to the actual TcMEP amplitude, suggesting that these changes did not significantly affect TcMEP amplitude. Using STS-INP during adolescent scoliosis surgery may enable accurate measurement of TcMEP amplitude without complications or influence of intraoperative factors.

## Figures and Tables

**Table 1:** Patient demographic, preoperative radiographic and laboratory, and intraoperative data (*n* = 50).

Demographic Data		Radiographic and Laboratory Data	
Age (years)	14 [13, 17]	Diagnosis	
Sex		Idiopathic scoliosis	41 (82%)
Female/Male	41 (82%)/9 (18%)	Symptomatic scoliosis	7 (14%)
Height (m)	1.59 [1.55, 1.63]	Congenital scoliosis	2 (4%)
Weight (kg)	48 [44, 54]	Cobb angle	
Body mass index (kg/m^2^)	18.92 [17.37, 21.30]	Main curve (°)	54 [50, 62]
Body surface area (m^2^)	1.44 [1.36, 1.52]	Bending main curve (°)	16 [11, 26]
ASA-PS class		Flexibility of main curve (%)	70 [56, 80]
1/2	39 (78%)/11 (22%)	Kyphosis (°)	14 [9, 23]
Complication		Hemoglobin (mg/dL)	12.20 [11.62, 13.10]
None	35 (70%)	Hematocrit (%)	37.00 [35.82, 39.77]
Chiari malformation	3 (20%)		
Marfan syndrome	3 (20%)	Intraoperative data	
ADHD	1 (6.7%)	Operation time (min)	252 [212, 283]
Autism	1 (6.7%)	Anesthesia time (min)	358 [305, 400]
Asthma	1 (6.7%)	Number of fused levels	10 [8, 11]
Depression	1 (6.7%)	Number of facetectomies	9 [7, 10]
Developmental disorder	1 (6.7%)	Total fluid intake (mL)	3000 [2278, 4068]
Kawasaki disease	1 (6.7%)	Total transfusion (mL)	295 [0, 648]
Migraine	1 (6.7%)	Urine output (mL)	532 [340, 1008]
Multiple osteochondroma	1 (6.7%)	Water balance (mL)	1765 [1178, 2304]
Renal dysfunction	1 (6.7%)		
Vital signs at admission			
Pulse rate (waves/min)	84 [74, 93]		
Mean NIBP (mmHg)	107 [99, 113]		

Data are expressed as numbers (percentage) or medians [interquartile range]. ASA-PS—American Society of Anesthesiologists—Performance Status; ADHD—attention deficit hyperactivity disorder; NIBP—noninvasive blood pressure.

**Table 2:** Stimulation settings and TcMEP amplitudes with STS-INP.

	All (*n* = 1412)	Age 10–14 Years (*n* = 760)	Age 15–19 Years (*n* = 652)
TcMEP stimulation settings			
Number of pulses	13 [11, 14]	13 [12, 15]	12 [11, 13]
Stimulation intensity (mA)	150 [140, 160]	150 [140, 170]	140 [130, 150]
TcMEP amplitude (mV)			
Abductor pollicis brevis muscle	3.25 [2.50, 4.18]	3,20 [2.44, 4.10]	3.30 [2.55, 4.30]
Tibialis anterior muscle	1.36 [0.89, 1.91]	1.16 [0.72, 1.59]	1.59 [1.12, 2.40]
Abductor hallucis muscle	1.96 [1.39, 2.70]	1.78 [1.18, 2.53]	2.15 [1.61, 2.80]

Data are expressed as medians [interquartile range]. TcMEP—transcranial motor-evoked potential; STS-INP—single-train stimulation with an increased number of pulses.

**Table 3:** Number of pulses and stimulus intensity in recent reports of spinal surgery, including scoliosis correction surgery.

Author	Patients	Number of Pulses	Stimulus Intensity
Ushirozako et al. [23]	Adult spinal deformity: 282	5	200 mA
	Adolescent idiopathic scoliosis: 100		
	Other types of scoliosis: 11		
Ando et al. [24]	Syndromic and neuromuscular scoliosis: 23	4 to 5	300 to 600 V
Neira et al. [25]	Pediatric scoliosis: 296	5	Not mentioned
Pastorelli et al. [26]	Congenital scoliosis/kyphoscoliosis: 15	5 to 7	up to 200 mA
	Adolescent idiopathic scoliosis/kyphoscoliosis: 76		
	Adult idiopathic scoliosis/kyphoscoliosis: 52		
	Other spine deformities: 29		
Kundnani et al. [27]	Adolescent idiopathic scoliosis (aged 8 to 18 years): 354	2 to 7	250 to 500 V

**Table 4:** Intraoperative factors affecting TcMEPs at the time of stimulation (*n* = 706).

Physiological factors	
Mean arterial blood pressure (mmHg)	55 [51, 61]
Percutaneous oxygen saturation (%)	100 [100, 100]
Core temperature (°C)	36.70 [36.40, 37.00]
Pulse rate (waves/min)	78 [69, 85]
Factors related to the depth of anesthesia	
Bispectral index	44 [39, 49]
Signal quality index	95.5 [94.2, 98.7]
Electromyogram activity (dB)	28.3 [27.5, 29.4]
Suppression ratio (%)	0 [0, 0]
Pharmacological factors	
Estimated blood concentration of propofol (µg/mL)	3.40 [3.00, 3.91]
Surgical factors	
Surgical elapsed time at TcMEP stimulation (min)	144 [98, 199]

Data are expressed as medians [interquartile range]. TcMEP—transcranial motor-evoked potential.

**Table 5:** Mixed-effect model with random intercept for transcranial motor-evoked potential amplitudes (mV).

	Abductor Pollicis Brevis Muscle Amplitude (*n* = 1412)	
	Regression Coefficient [95% CI]	Predicted Mean Difference [95% CI *]
Mean arterial blood pressure (mmHg)	0.004 [−0.005, 0.012]	0.036 [−0.049, 0.121]
Bispectral index	0.003 [−0.007, 0.014]	0.033 [−0.070, 0.136]
Estimated blood concentration of propofol (µg/mL)	−0.217 [−0.501, 0.068]	−0.198 [−0.456, 0.062]
Core temperature (°C)	−0.140 [−0.432, 0.150]	−0.084 [−0.259, 0.090]
Oxygen saturation (%)	0.122 [−0.027, 0.269]	**
Number of pulses (times)	0.035 [−0.039, 0.108]	0.105 [−0.117, 0.325]
Stimulus intensity (×10 mA)	−0.076 [−0.171, 0.019]	−0.153 [−0.343, 0.039]
Surgical elapsed time at TcMEP stimulation (min)	0.000 [−0.001, 0.002]	0.050 [−0.095, 0.197]
	Tibialis Anterior Muscle Amplitude (*n* = 1412)	
	Regression Coefficient [95% CI]	Predicted Mean Difference [95% CI *]
Mean arterial blood pressure (mmHg)	−0.005 [−0.012, 0.002]	−0.054 [−0.124, 0.016]
Bispectral index	0.003 [−0.006, 0.012]	0.030 [−0.055, 0.115]
Estimated blood concentration of propofol (µg/mL)	−0.031 [−0.285, 0.217]	−0.028 [−0.259, 0.198]
Core temperature (°C)	0.185 [−0.057, 0.431]	0.111 [−0.034, 0.258]
Oxygen saturation (%)	0.080 [−0.042, 0.204]	**
Number of pulses (times)	−0.074 [−0.138, −0.012]	−0.223 [−0.413, −0.036]
Stimulus intensity (×10 mA)	−0.044 [−0.128, 0.038]	−0.088 [−0.256, 0.077]
Surgical elapsed time at TcMEP stimulation (min)	−0.001 [−0.002, 0.001]	−0.055 [−0.176, 0.066]
	Abductor Hallucis Muscle Amplitude (*n* = 1412)	
	Regression Coefficient [95% CI]	Predicted Mean Difference [95% CI *]
Mean arterial blood pressure (mmHg)	−0.003 [−0.011, 0.004]	−0.035 [−0.107, 0.037]
Bispectral index	0.005 [−0.004, 0.013]	0.045 [−0.043, 0.133]
Estimated blood concentration of propofol (µg/mL)	−0.036 [−0.289, 0.216]	−0.033 [−0.263, 0.196]
Core temperature (°C)	0.118 [−0.132, 0.370]	0.071 [−0.079, 0.222]
Oxygen saturation (%)	−0.082 [−0.208, 0.044]	**
Number of pulses (times)	−0.034 [−0.099, 0.031]	−0.103 [−0.297, 0.092]
Stimulus intensity (×10 mA)	0.126 [0.040, 0.210]	0.251 [0.080, 0.420]
Surgical elapsed time at TcMEP stimulation (min)	−0.001 [−0.003, −0.000]	−0.137 [−0.262, −0.013]

* confidence interval (CI) for the predicted mean difference evaluated using the interquartile range of each factor. ** data were unsuitable for analysis because the IQR had zero.

## Data Availability

The datasets used and/or analyzed during the current study are available from the corresponding author on reasonable request.
